# Extending the culture duration could not improve the culture positivity rate and clinical outcomes of periprosthetic joint infection

**DOI:** 10.3389/fcimb.2025.1551862

**Published:** 2025-05-08

**Authors:** Baijian Wu, Yuan Lin, Jinhui Su, Lan Lin, Zhenggui Yu, Chaofan Zhang, Xinyu Fang, Zida Huang, Wenming Zhang

**Affiliations:** ^1^ Department of Orthopaedic Surgery, National Regional Medical Center, Binhai Campus of the First Affiliated Hospital, Fujian Medical University, Fuzhou, China; ^2^ Department of Orthopaedic Surgery, The First Affiliated Hospital, Fujian Medical University, Fuzhou, China; ^3^ Department of Orthopaedic Surgery, Zhongshan Hospital of Xiamen University, Xiamen, China

**Keywords:** periprosthetic joint infection, culture period, time to positivity, pathogens, hip & knee arthroplasty

## Abstract

**Objective:**

To explore the clinical value of extending the culture time for accurately diagnosing hip or knee PJI.

**Methods:**

This retrospective study included 201 hip or knee PJI patients. All specimens were cultured using a standardized procedure. For all samples collected after January 2022, the extended culture period was 14 to 21 days. The detection accuracy and pathogen distribution of the standard culture duration (7 days) was compared with those extending.

**Results:**

The overall culture positivity rate was 89.05% (179/201). The most common pathogens were coagulase-negative staphylococci (CoNS, n=54) and methicillin-resistant *Staphylococcus aureus* (MRSA, n=26). Extending the culture duration did not significantly increase the culture positivity rate (89.05% vs. 89.06%, P=0.997), nor did it improve the infection control rate (89.05% vs. 85.94%, P=0.526). Further study revealed that extended results did not improve the diagnosis of PJI according to the Musculoskeletal Infection Society (MSIS) criteria in most patients with both positive standard and extended culture results (82.35%, 28/34). Four of the 5 patients with only positive extended culture results were diagnosed with PJI on the basis of a single positive culture result.

**Conclusion:**

Extending the culture time didn’t significantly improve the clinical outcomes of PJI, rate of culture positivity or polymicrobial infection detection rate.

## Introduction

With the widespread implementation of total joint arthroplasty, the incidence of periprosthetic joint infection (PJI) has been increasing progressively. PJI is a serious complication of total joint arthroplasty and pathogen diagnosis is considered the “gold standard” for the diagnosis of PJI. The Musculoskeletal Infection Society (MSIS) diagnostic criteria state that two positive periprosthetic cultures with phenotypically identical organisms can serve as the primary criterion for PJI diagnosis; conversely, a single positive culture is considered a secondary criterion for the diagnosis of this disease (Parvizi and Gehrke, 2014). The identification of pathogens not only clarifies the diagnosis of PJI but also guides the appropriate use of antibiotics. Despite studies suggesting that the clinical outcomes of culture-negative PJI (CNPJI) are not inferior to those of culture-positive PJI and that negative microbiological results are not risk factors for failure to control infection (Kang et al., 2018; Li et al., 2023; Xu et al., 2022), the incidence of antibiotic side effects is indeed greater in culture-negative PJI (Xu et al., 2022; Lai et al., 2024).

Identifying the etiological pathogen is crucial, yet there is currently no consensus regarding the best duration of culture (Schäfer et al., 2008). The International Consensus Meeting (ICM) recommends (Birlutiu et al., 2022; Parvizi et al., 2013; Ascione et al., 2019) that a minimum of four intraoperative culture samples should be collected to increase the rate of culture positivity. Cultures should be maintained for 5–7 days; in cases of suspected PJI caused by low-virulence microorganisms or cases where preoperative cultures are negative but there is a high degree of clinical suspicion of PJI, the culture duration should be extended to 14–21 days. However, recent studies on the duration of PJI culture have provided conflicting information (Birlutiu et al., 2022; Klement et al., 2019; Talsma et al., 2021). Birlutiu et al (Birlutiu et al., 2022). noted that extending the culture incubation period (to more than 14 days) in chronic PJI does not further increase the ability to identify microorganisms. Catalina et al. also found that fourteen-day cultures did not increase the positivity rate, rate of polymicrobial infections, or number of positive specimens reported (Baez et al., 2024).

The replication of pathogens is a complex process influenced by various factor. Pathogens that are contaminated, have low virulence, or have formed mature biofilms are more difficult to cultivate (Schäfer et al., 2008; Wood et al., 2013). Previous studies have used methods such as blood culture vials and isothermal microcalorimetry to accelerate the identification of pathogens (Minassian et al., 2014; Cichos et al., 2023). In this study, we systematically evaluated the effect of different culture period on the detection of pathogens in hip and knee PJI, aiming to reveal the significance of solely extending the culture period for improving the accurate detection of PJI.

## Method

Ethical approval was obtained from the First Affiliated Hospital of Fujian Medical University Ethics Committee. (approval no. MRCTA, ECFAH of FMU (2019) 296). This study was a single-center, retrospective consecutive study that included 201 patients (103 patients with hip PJI and 98 patients with knee PJI) who were diagnosed with PJI from January 2013 to March 2024. The diagnosis of PJI met the MSIS diagnostic criteria^4^. Patients for whom attempts had been made to improve pathogen detection via alternative methods other than extended culture during pathogen culture were excluded. We used a standard culture period until January 2022, after which all samples were subjected to extended culture. The demographic data, infection types and clinical outcomes of these patients were collected, along with the type of specimens, time to positivity (TTP) and microbiological outcomes with positive culture results (Fang et al., 2021b). Negative standard cultures were defined as specimens that had been cultured for 7 days without pathogen growth. Extended cultures were defined as a specimen culture period of 14 to 21 days.

In accordance with the recommendations of the MSIS, infection control was assessed one year after the initiation of PJI treatment, considering that reinfections occurring more than one year after the start of PJI treatment are unlikely to represent a failure of the initial treatment (Fillingham et al., 2019).

### Standardized protocol for pathogen acquisition and culture

A set of standardized pathogen acquisition and culture procedures were performed at our institution. All patients discontinued antibiotic therapy for more than two weeks prior to receiving PJI management. Routine joint aspiration under ultrasound guidance was performed preoperatively following strict aseptic procedures. At least 3 mL of joint fluid (JF) was collected for microbiological culture. The JF strains were smeared on Columbia blood agar media (Haibo Biotechnology, Qingdao, China) and cultured under aerobic and anaerobic conditions. The remaining samples were immediately inoculated into BACTEC Plus/F aerobic and anaerobic vials (Becton-Dickinson, Franklin Lakes, New Jersey, USA) and cultured in a BACTEC 9050 automated culture system (FX 400; Becton Dickinson) for six days before being removed and subcultured, which has been considered feasible and beneficial in previous studies (Ascione et al., 2019; Beguiristain et al., 2023; Azad and Patel, 2024).

Our institution routinely performs sonication on explanted prostheses (Huang et al., 2020; Fang et al., 2021a; Wang et al., 2020). The JF, periprosthetic tissue (PT), and sonication fluid (SF) obtained intraoperatively were transported to the clinical microbiology laboratory by the same epidemiologist within 30 minutes and cultured in BACTEC Plus/F vials and on Columbia blood agar media immediately. The results of the SFC were determined by both the surgeon and the epidemiologist to rule out contamination, and a positive result was considered to be > 50CFU/mL of any microorganism for uncentrifuged samples (Trampuz et al., 2007; Rondaan et al., 2023).

### Statistical analysis

Continuous data are presented as means and standard deviations, or medians and 25th and 75th percentiles, depending on the distribution. Statistical differences between groups were compared using the Student’s t-test or the rank-sum test. Categorical data were represented by counts and percentages, and comparisons between groups were made using the chi-square test, Fisher’s exact test or Fisher-Freeman-Halton exact test. A p-value of less than 0.05 was considered to indicate statistical significance. All analyses were conducted using SPSS version 26.0 (IBM, Armonk, New York, USA). The statistical graph was produced by praph-pad 9.5.1.

## Results

In total, 201 patients were included in this study. All patients were divided into a standard group (n=137) and an extension group (n=64) according to the duration of the pathogen culture. The demographic data and clinical outcomes of the two groups are shown in **Table 1**. In the standard group, 95 patients had multiple positive culture results, 27 patients had a single positive culture result. There were also 40 and 12 of these patients, respectively, in the extension group. The overall culture positivity rate was 89.05% (179/201). The percentage of standard positive cultures was 89.05% (122 of 137), and extending the culture period did not significantly increase pathogen detection (89.05% vs. 89.06%, P=0.997). The two groups had 11 and 8 cases of polymicrobial infections, respectively, which was not significantly different. In terms of clinical outcomes, the overall infection control rates were 88.06% (177 of 201). Extended culture did not improve the clinical outcomes of PJI patients (P=0.526).

**Table 1:** Demographic data and clinical outcomes.

Variables	Standard group (n=137)	Extension group (n=64)	*P* value
Sex (%)			0.947^a^
Male	60(43.80)	28(43.75)	
Female	77(56.20)	36(56.25)	
Age, yr	65.52±11.30	65.20±11.76	0.878^b^
BMI, kg/m^2^	24.54±3.26	25.65±3.50	0.133^b^
Sample size (%)			0.621^a^
Knee	65(47.45)	33 (51.56)	
Hip	72(52.55)	31 (48.44)	
Tsukuyama type (%)			0.935^a^
Acute or hematogenous	35(25.55)	16(25.00)	
Chronic	102(74.45)	48(75.00)	
Positive culture rate (%)			0.997^a^
Culture positive	122(89.05)	57 (89.06)	
CNPJI	15(10.95)	7(10.94)	
Polymicrobial infection (%)			0.250^a^
Yes	11(8.03)	8(12.50)	
No	126(91.97)	56(87.50)	
Infection control (%)			0.526^a^
Yes	122(89.05)	55 (85.94)	
No	15(10.95)	9(14.06)	

BMI, body mass index; CNPJI, culture-negative periprosthetic joint infection.

Data presented as mean ± SD or as count (percentage).

a, chi-square test.

b, Student's t-test.

There was no significant difference in the distribution of pathogens between the two groups (**Table 2**). The most common culture-positive pathogens were coagulase-negative *staphylococci* (CoNS, n=54) and methicillin-resistant *Staphylococcus aureus* (MRSA, n=26). Notably, in this study, *Cutibacterium acnes* (*C. acnes*) was detected in most samples subjected to standard culture, whereas only one case of *C. acnes* infection was detected in the extension group. Two PT samples from this patient were found to be infected with *C. acnes*, meeting the MSIS primary criteria. However, pathogen growth was detected in one sample at 96.7 hours after acquisition, whereas the TTP of another sample was 255.75 hours. This means that some specimens in the extension group may have been detected within 168 hours (7 days) and that the extended culture results were still negative.

**Table 2:** Pathogen results.

Variables	Standard group (n=137)	Extension group (n=64)	Total	*P* value
*Staphylococcus aureus*	18 (13.14)	5 (7.81)	23 (11.44)	
MRSA	17 (12.41)	9 (14.06)	26 (12.94)	
CoNS	37 (27.01)	17 (26.56)	54 (26.87)	
*Streptococcus*	10 (7.30)	8 (12.50)	18 (8.96)	
*Enterococcus*	4 (2.92)	2 (3.13)	6 (2.99)	
Gram-negative *bacilli*	12 (8.76)	6 (9.38)	18 (8.96)	
*Cutibacterium acnes*	4 (2.92)	1 (1.56)	5 (2.49)	
Fungal	3 (2.19)	1 (1.56)	4 (1.99)	
Others	6 (4.38)	0 (0)	6 (2.99)	
Polymicrobial	11 (8.03)	8 (12.50)	19 (9.45)	
Culture-negative	15 (10.95)	7 (10.94)	22 (10.95)	
				0.999^b^

MRSA, methicillin-resistant *Staphylococcus aureus*; CoNS, coagulase negative *staphylococci*.

a, Others included *Corynebacterium striatum*, *Micrococcus luteus*, *Burkholderia cepacian*, *Finegoldia magna*, *Clostridium perfringen*, *Mycobacterium Foruitum* and *Mycobacterium Tuberculosis*.

b, Fisher's exact test.

**Figure 1:**
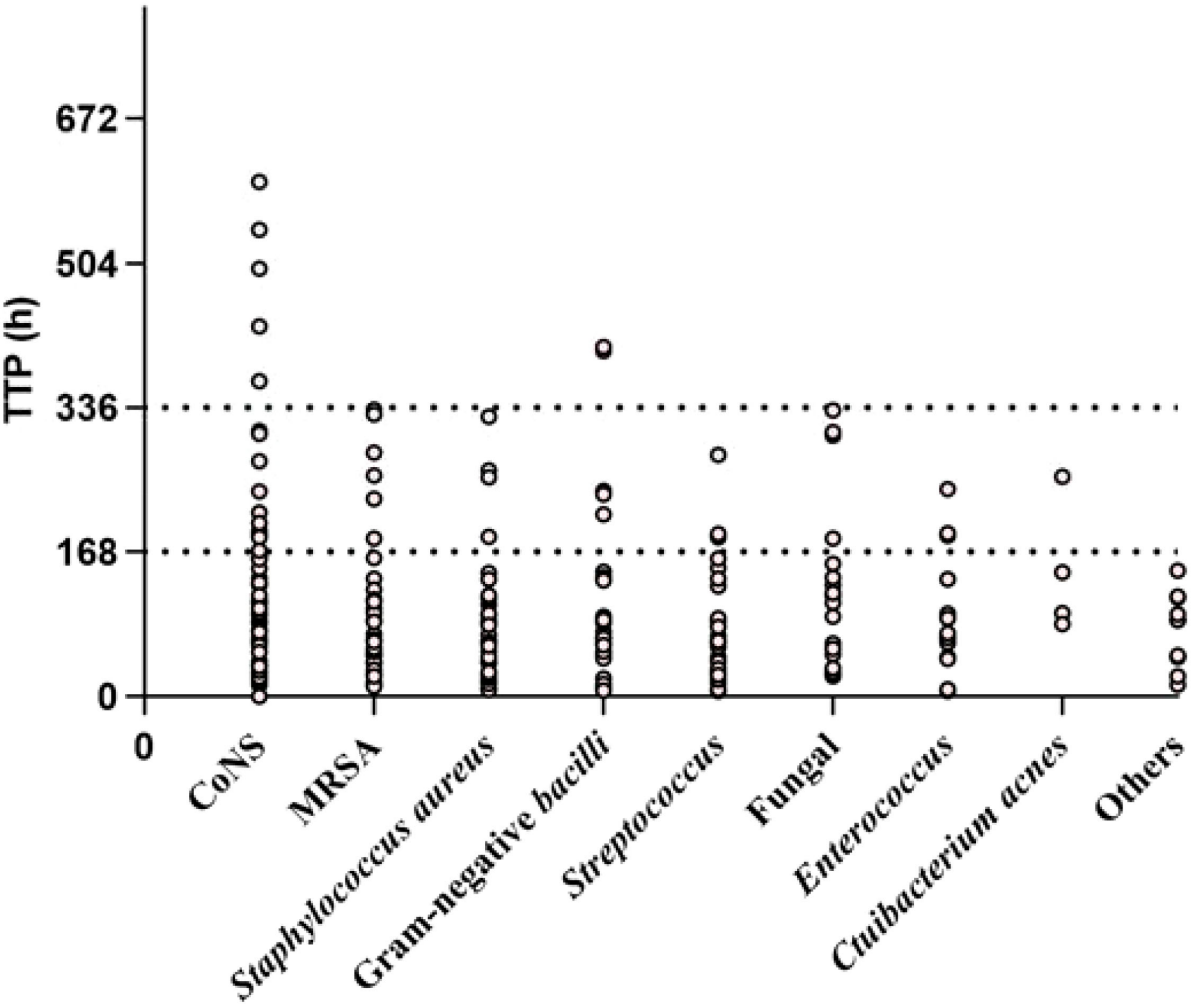
Culture trends of different pathogens TTP, time to positive.

**Figure 2:**
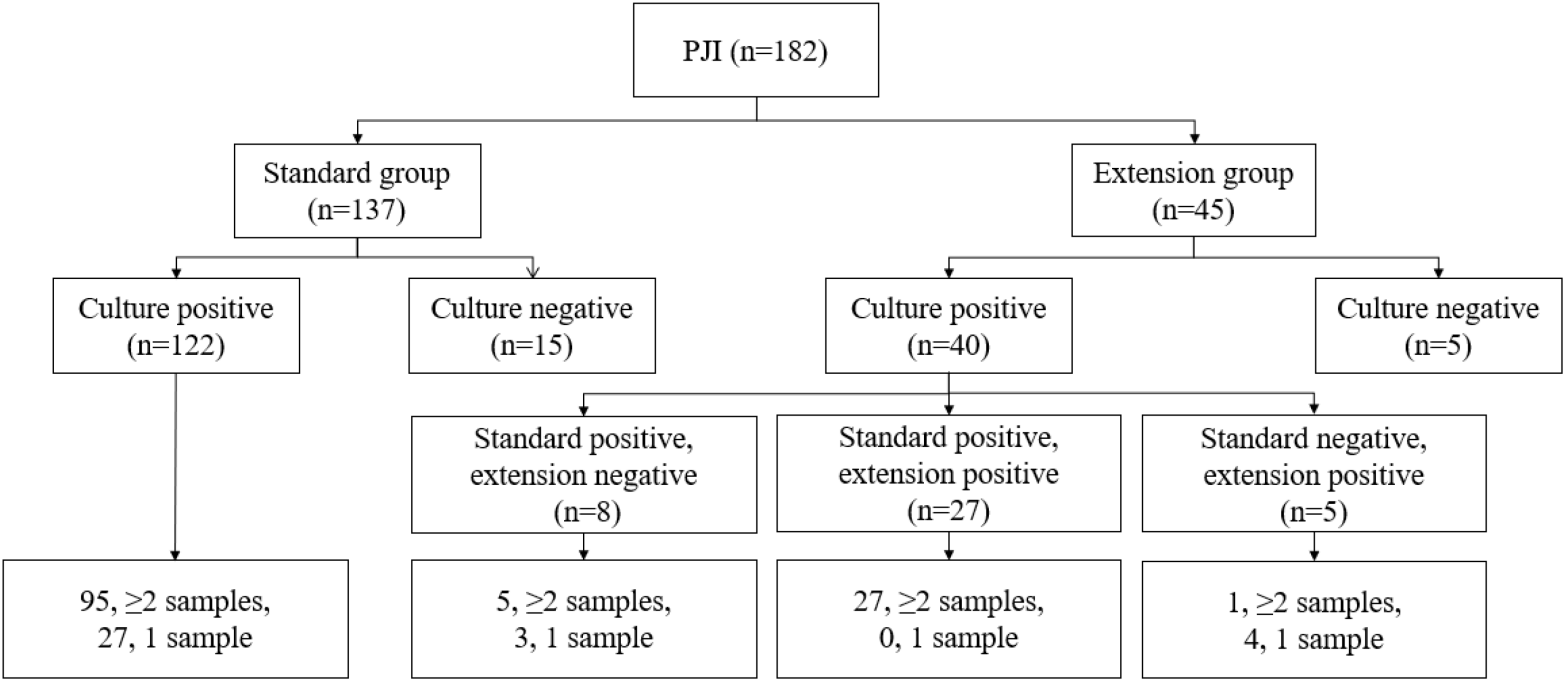
Distribution of cases according to the various culture results.

**Figure 3:**
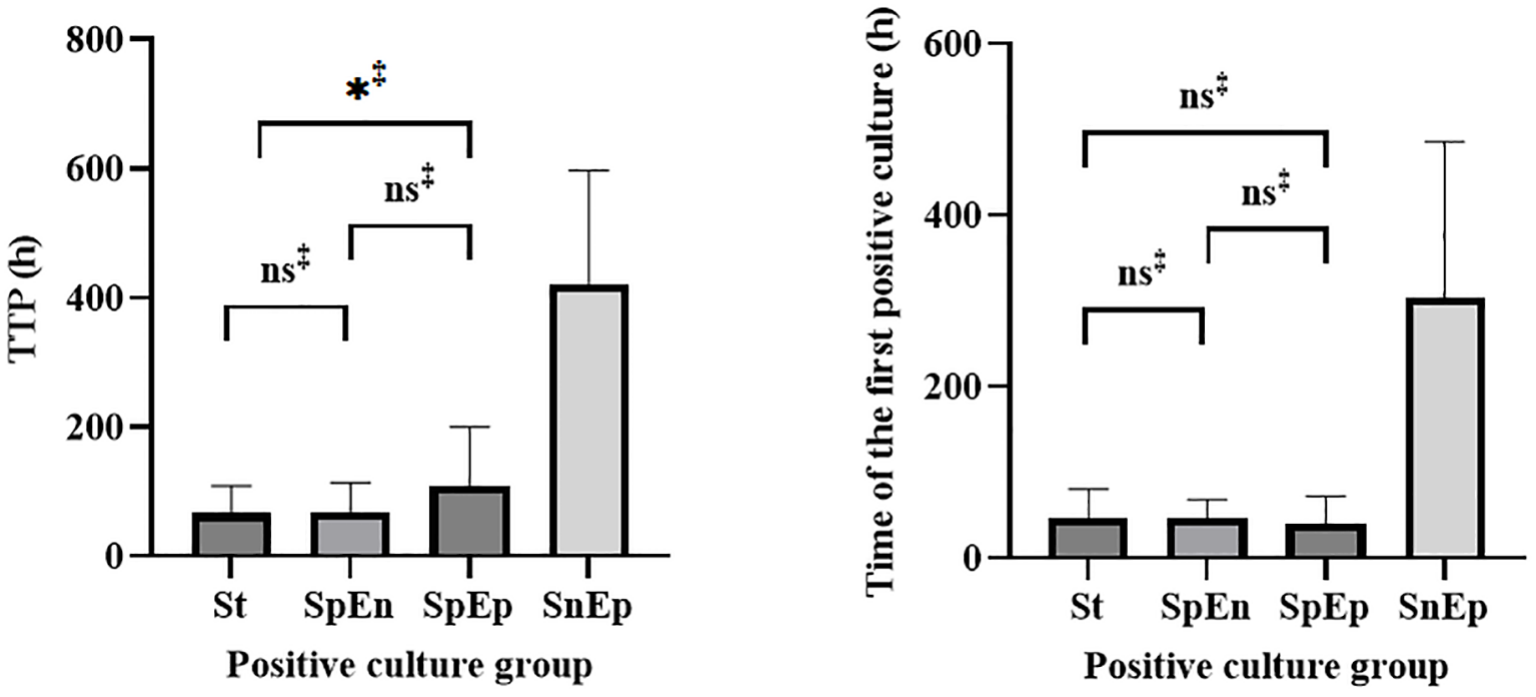
Statistical analysis of the TTP and time of first positive detection in the standard, SpEn, and SpEp groups.St= standard group.‡, Mann–Whitney U test.

Pathogens were identified within the standard time range in most cases of culture-positive PJI (97.21%, 174 of 179). In the extension group, the patients with positive cultures were further classified according to the extended results (**Figure 1**), including standard positive and prolonged negative tests (SpEn, n=18), standard positive and prolonged positive tests (SpEp, n=34), and standard negative and prolonged positive tests (SnEp, n=5). We then compared the number of positive samples, TTP, and time to first positive culture report among patients with culture-positive PJI (**Table 3**). The TTP and time to the first positive detection of SnEp were much longer than those in the other three groups (P < 0.001). Although the results revealed significant differences in the number of positive samples in the different groups, this difference was due to the group setting. All TTPs and times to the first positive detection of SnEp were much longer than those in the other three groups (P< 0.001). The different culture trends of different pathogens are presented in detail in **Figure 2**. In the standard group, SpEn group and SpEp group, there was no statistically significant difference between the time of the first positive detection, whereas all TTPs in the standard group were significantly lower than those in the SpEp group (**Figure 3**). The data for the SpEp group and SnEp group are shown in **Appendix Table 1**. The extended results did not interfere with the diagnosis of PJI. The pathogens in the SnEp group included 2 cases of CoNS, 1 case of gram-negative *bacilli*, 1 case of MRSA, and 1 case of *Streptococcus* (**Appendix Table 1**). We subsequently reviewed the sample of pathogens. Multiple CoNS were identified from intraoperative JF and PT (No.38 in **Appendix Table 1**). The isolated gram-negative *bacilli* and *Streptococci* were obtained from intraoperative PT and intraoperative JF, respectively. MRSA and another isolated CoNS were obtained from intraoperative SF. In this present study, there were differences in the culture positive rates of PT and JF in different groups (P_PT_=0.002, P_JF_=0.001, **Table 3**).

**Table 3:** Detailed culture results for patients with positive culture.

Variables	Standard group(n=122)	Extension group			*P* value
		SpEn(n=18)	SpEp(n=34)	SnEp(n=5)	
Positive culture samples (%)					<0.001^a^
≥2 samples	95 (77.87)	12 (66.67)	27 (100)	1 (20)	
1 sample	27 (22.13)	6 (33.33)	0 (0)	4 (80)	
All TTPs (h)	64.57 (28.30, 91.00)	65.03 (34.63, 117.38)	74.85 (38.26, 173.96)	508.16 (187.36, 556.78)	<0.001^b^
Time of the first positive culture (h)	40.46 (15.89, 68.56)	48.33 (27.60, 69.67)	38.57 (13.38, 59.82)	188.75 (183.18, 482.03)	<0.001^b^
Sample type (%)					
PT	62 (50.82)	9 (50.00)	21 (61.76)	2 (40.00)	0.002^c^
JF	82 (67.21)	12 (66.67)	25 (73.53)	2 (40.00)	0.001^c^
SF	49 (40.16)	8 (44.44)	15 (44.11)	2 (40.00)	0.901^c^

TTP, time to positive; SpEn, standard positive & extension negative; SpEp, standard positive & extension positive; SnEp, standard negative & extension positive; PT, periprosthetic tissue; JF, joint fluid; SF, sonicated fluid.

a, Fisher's exact test.

b, Kruskal-Wallis H Test.

c, Fisher-Freeman-Halton exact test.

## Discussion

Pathogen detection is an important basis for the diagnosis and treatment of PJI. In previous reports, surgeons and infectologists have adopted various approaches to optimize the culture process in an attempt to improve pathogen detection in PJI (Schäfer et al., 2008; Trampuz et al., 2007; Otero et al., 2024). Moreover, in PJI cases that are suspected to be caused by low-virulence microorganisms or that have negative preoperative cultures, prolonging the culture time may also help to improve the rate of its culture positivity (Parvizi et al., 2019). The total rate of positivity was 89.05% (179/201) in this study, which was similar to that in a previous report (Parvizi et al., 2014). In recent years, some studies have explored pathogen culture according to the timing of PJI. Talsma et al. reported pathogen growth in acute and chronic PJI within 5 and 11 days, respectively (Talsma et al., 2021). Although these studies emphasized the application of extended culture in PJIs, they excluded CNPJI without exception (Schäfer et al., 2008; Birlutiu et al., 2022; Talsma et al., 2021). In this study, we included all culture-negative cases to explore the clinical application of this strategy in the management of PJI.

The results revealed that extended culture did not help reduce the rate of CNPJI, increase the diagnosis of polymicrobial infections, or improve the clinical outcome of patients. Klement et al. compared the duration of culture in 189 patients with acute PJI and obtained similar results (Klement et al., 2019). In our cohort, *C. acnes* was grown in all five patients within standard times. Only one sample was identified during the extended period. On the basis of previous reports, blood culture bottles appear to have an advantage in their ability to detect *C. acnes*. Minassian et al. used BACTEC™ culture to detect PJI pathogens and noted that most bacteria (including *C. acnes*) could be identified and did not require extension to 2 weeks (Minassian et al., 2014). Another study also showed that blood culture bottles can detect pathogenic bacteria significantly faster (Birdsall et al., 2021). The majority of pathogens (97.21%, 174 of 179) were isolated within the standard time, and the TTP of the first sample was not significantly different, which was similar to previous studies (Klement et al., 2019; Schwotzer et al., 2014). Schwotzer et al. reviewed 58 cases of infectious orthopedic revision surgery, in which 96.6% (56 in 58) of the infections were diagnosed within 7 days (Otero et al., 2024). Notably, although all SpEp patients in that cohort met the major MSIS criteria, when the extended results were removed, only 17.65% (6 in 34) of the patients had a single positive specimen within the standard time. When these criteria were combined with the remaining criteria (minor criteria or sinus tracts), the patients were still confirmed as having PJI. Positive culture results obtained within the standard times were considered clinically significant.

At present, the most convincing argument for extending the duration of culture in cases of hip and knee PJI is the difficulty in identifying fastidious bacteria, such as some gram-negative bacilli (Berbari et al., 2007; Tarabichi et al., 2023) and small colony variants (SCVs). Frequently, these slowly developing phenotypic variants go unnoticed or are incorrectly identified because of their atypical physical characteristics and distinctive biochemical responses. Tuchscherr et al. reported that almost all SCVs isolated from clinical specimens can revert to the parental state and grow rapidly (Tuchscherr et al., 2020). Adjustment to an appropriate growth environment (sensitive supplemented growth media, oxygen, humidity, etc.) could promote the transformation of pathogenic bacteria and shorten the time limit of culture. When low-virulence fastidious bacteria are suspected to be the cause of infection, the culture environment should be optimized early to promote bacterial growth. According to the results of preoperative next-generation sequencing (NGS), Fang et al (Beguiristain et al., 2023). provided unique media or culture conditions for pathogenic bacteria and improved the identification efficiency. In our study, three cases of *Staphylococcus* and one case of gram-negative bacilli were included in the SnEp group. However, extended culture results presented a challenge to our work, although such cases were rarely observed in this study (2.49%, 5 in 201). In our study, most of the patients in the SnEp group had PJIs that yielded a positive single culture. The diagnosis of a single culture positive sample is challenging, although our patients all meet the MSIS criteria. Rondaan et al. pointed out that patients with a simple positive SF culture were more likely to develop infection (Rondaan et al., 2023), which means that when a single culture specimen does not meet current diagnostic criteria, perhaps combined with other means, such as molecular diagnostics (Beguiristain et al., 2023), can improve the persuasive strength of these samples, which needs further study. For tiny colonies that are difficult to identify in the medium, it might be possible to promote the transformation and rapid growth of pathogens through early optimization of the culture environment. We recommended an extended culture period combined with other optimized methods to improve PJI management.

This study has several limitations. First, as a retrospective study, the number of included studies was limited, and the number of *C. acnes* organisms in the extended group was small, which may have increased the difficulty in obtaining correct results. Second, there was a lack of consistency in the samples in the present study, with different types of specimens obtained by preoperative aspiration, intraoperative sampling, and postoperative processing, although this was necessary for the management of PJI. Homogenization is meaningful when comparing the benefits of culturing samples from different sources. Although our study found low positive rates of various specimen cultures in the SnEp group, considering such a sparse number reduces the reliability of the results. The type of specimen may have an effect on the culture cycle, which needs further study. Third, we did not consider the burden of work caused by unconditional extension of culture. Therefore, it would be valuable to perform a prospective study to compare the benefits of diagnosing pathogens by culturing tissue samples of different stages and types to improve culture efficiency and reduce the abovementioned burden as much as possible.

## Conclusion

Our results showed that solely extending the culture period led to the detection of some pathogens and did not significantly improve the clinical outcomes of PJI, rate of culture positivity or polymicrobial infection detection rate.

## Data Availability

The raw data supporting the conclusions of this article will be made available by the authors, without undue reservation.
