# Supplementary material for: Extending the culture duration could not improve the culture positivity rate and clinical outcomes of periprosthetic joint infection
Source: Front Cell Infect Microbiol. 2025 May 8;15:1551862. doi: 10.3389/fcimb.2025.1551862 (PMC12095319; doi:10.3389/fcimb.2025.1551862)
Supplement: Supplementary file 1 [file Table1.pdf]

Appendix Table 1. MSIS criteria in SpEp group and SnEp group

Patient number	Tsukuyama type	Pathogen	Major criteria			Minor criteria					
			Sinus tract	Multiple pathogens	Multiple pathogens[c]	Elevated ESR and CRP	Elevated synovial WBC count	Elevated PMN percentage	Purulence	Intraoperative findings of positive histology	Single positive culture
1[a]	Chronic	MRSA	No	Yes	Yes	Yes	Yes	Yes	No	No	Yes
2[a]	Chronic	*Enterococcus*	No	Yes	Yes	Yes	Yes	No	No	No	Yes
3[a]	Acute	Fungal	No	Yes	Yes	No	Yes	No	No	Yes	Yes
4[a]	Hematogenous	MRSA	No	Yes	Yes	Yes	Yes	No	No	No	Yes
5[a]	Chronic	MRSA	Yes	Yes	Yes	Yes	No	No	Yes	No	Yes
6[a]	Hematogenous	CoNS	No	Yes	Yes	Yes	Yes	Yes	No	Yes	Yes
7[a]	Chronic	*Streptococcus*	No	Yes	Yes	Yes	Yes	Yes	No	Yes	Yes
8[a]	Chronic	CoNS	No	Yes	Yes	Yes	Yes	Yes	Yes	Yes	Yes
9[a]	Chronic	CoNS	No	Yes	Yes	Yes	Yes	Yes	No	Yes	Yes
10[a]	Chronic	CoNS	Yes	Yes	Yes	Yes	No	No	Yes	Yes	Yes
11[a]	Chronic	*Staphylococcus aureus*	Yes	Yes	Yes	Yes	Yes	Yes	No	Yes	Yes
12[a]	Acute	*Propionibacterium acnes*	Yes	Yes	No	Yes	No	No	Yes	Yes	Yes
13[a]	Chronic	Polymicrobial	No	Yes	Yes	Yes	No	Yes	Yes	No	Yes
14[a]	Chronic	CoNS	No	Yes	Yes	Yes	Yes	Yes	Yes	Yes	Yes
15[a]	Chronic	Polymicrobial	No	Yes	Yes	Yes	Yes	Yes	No	Yes	Yes
16[a]	Hematogenous	*Staphylococcus aureus*	No	Yes	Yes	No	Yes	Yes	No	Yes	Yes
17[a]	Hematogenous	Gram-negative *bacilli*	No	Yes	Yes	No	Yes	No	Yes	Yes	Yes
18[a]	Chronic	MRSA	No	Yes	Yes	Yes	No	No	Yes	Yes	Yes
19[a]	Chronic	CoNS	Yes	Yes	No	Yes	No	Yes	Yes	Yes	Yes
20[a]	Chronic	*Streptococcus*	No	Yes	No	Yes	Yes	Yes	No	No	Yes
21[a]	Chronic	Polymicrobial	No	Yes	Yes	Yes	Yes	Yes	Yes	No	Yes
22[a]	Chronic	MRSA	No	Yes	No	Yes	No	No	Yes	Yes	Yes
23[a]	Chronic	Gram-negative *bacilli*	Yes	Yes	Yes	Yes	No	Yes	Yes	Yes	Yes
24[a]	Chronic	*Staphylococcus aureus*	No	Yes	Yes	Yes	Yes	Yes	Yes	Yes	Yes
25[a]	Chronic	Polymicrobial	No	Yes	Yes	Yes	No	No	Yes	Yes	Yes
26[a]	Chronic	CoNS	Yes	Yes	Yes	No	Yes	No	Yes	No	Yes
27[a]	Chronic	Gram-negative *bacilli*	No	Yes	No	Yes	Yes	Yes	No	Yes	Yes
28[b]	Chronic	CoNS	No	No	-	Yes	Yes	Yes	No	Yes	Yes
29[b]	Chronic	Gram-negative *bacilli*	No	No	-	Yes	No	Yes	Yes	Yes	Yes
30[b]	Chronic	*Streptococcus*	No	No	-	Yes	Yes	Yes	No	Yes	Yes
31[b]	Chronic	CoNS	No	Yes	-	Yes	Yes	Yes	Yes	No	Yes
32[b]	Chronic	MRSA	Yes	No	-	No	Yes	No	Yes	Yes	Yes

ESR= erythrocyte sedimentation rate; CRP= C-reaction protein; WBC= white cell count; MRSA= methicillin-resistant Staphylococcus aureus, CoNS= coagulase negative staphylococci.

a, SpEp group

b, SnEp group

c, pathogen results without extended culture
